# Assisted reproductive technology and the risk of fetal congenital heart disease: insights from a tertiary-care referral center

**DOI:** 10.1007/s00404-024-07669-x

**Published:** 2024-08-01

**Authors:** Linda Piemonti, Laura Vettor, Anna Balducci, Antonio Farina, Elena Contro

**Affiliations:** 1 Department of Obstetrics and Gynecology, Obstetric Unit, IRCCS Azienda Ospedaliero-Universitaria Di Bologna, Via Giuseppe Massarenti, 13, 40138 Bologna, Italy; 2 Department of Women’s and Children’s Health Gynecologic and Obstetrics Clinic, University of Padua, Pauda, Italy; 3 Pediatric Cardiology Unit, IRCCS Azienda Ospedaliero-Universitaria Di Bologna, Bologna, Italy

**Keywords:** Congenital heart defects, Assisted reproductive technology, Intracytoplasmic sperm injection, In vitro fertilization, Fetal

## Abstract

**Purpose:**

To investigate whether congenital heart diseases exhibit higher rates in pregnancies achieved through assisted reproductive technology (ART) compared to natural conception.

**Methods:**

In this retrospective cohort study, multinomial logistic regression was employed to analyze the relationship between categories of congenital heart diseases and three conception groups (IVF, ICSI, and natural pregnancies). The main outcome measures are risks of congenital heart disease categories in IVF and ICSI groups using the natural group as reference. We selected fetuses referred for fetal echocardiography to IRCCS Policlinico Sant’Orsola, Bologna, between January 2005 and November 2023, diagnosed with congenital heart diseases.

**Results:**

We categorized the congenital heart diseases into six groups based on anatomical and embryological criteria. The estimated risk of left ventricular outflow tract, valvular, conotruncal, and atrioventricular septal defects was lower in the IVF group compared to natural conception. The estimated risk of valvular and atrioventricular septal defects was lower in the ICSI group vs natural. Conversely, the risk for right heart anomalies was higher both in the IVF and ICSI groups compared to natural conception. Heart rhythm diseases were more frequent in IVF pregnancies. When comparing ART methods, valvular defects, conotruncal defects, and right heart anomalies were more frequently observed in the ICSI group, while atrioventricular septal defects were more common in the IVF group.

**Conclusion:**

Significant differences were found in the occurrence of congenital heart diseases in pregnancies conceived through IVF and ICSI, versus those conceived naturally, underscoring the importance of further studying the underlying mechanisms of these associations.

## What does this study adds to the clinical work

**Table:** 

This study reveals significant differences in the occurrence of specific congenital heart disease categories between pregnancies conceived through ART and those conceived naturally. These findings underscore the need for further research to understand the mechanisms linking ART to CHD development.	

## Introduction

Congenital heart defects (CHD) stand as the most prevalent birth abnormalities and constitute a leading cause of infant mortality. Variations in definitions, population characteristics, and diagnostic methods result in a wide range of prevalence estimates. In Europe, the average total prevalence of CHD is 8.0 per 1000 births, with a live birth prevalence of 7.2 per 1000 births, varying between countries [1]. The utilization of assisted reproductive technology (ART) has experienced a significant rise in recent years, particularly with the adoption of oocyte and sperm donation. In the United States, approximately 2 percent of all births result from ART (ranging from 0.4% in Puerto Rico to 5.1% in Massachusetts) [2], while Italy has experienced a significant increase in the rate of ART-conceived births over the past fifteen years, reaching 3 percent of all births in 2022 [3]. It is well-established that conception through ART is associated with obstetric and perinatal complications, including multiple gestations, low birth weight (LBW), pre-term delivery [4], and congenital anomalies. Indeed, it has been consistently shown that the risk of congenital anomalies is potentially increased by approximately 60% compared to pregnancies conceived naturally [5]. However, uncertainties persist regarding the risks of birth defects, especially CHD. Particularly, the incidence of CHD differs greatly from study to study. The incidence of moderate and severe forms of CHD is approximately 6 per 1000 live births. Though, the overall incidence increases to 75 per 1000 live births if minor forms, such as tiny muscular ventricular septal defects (VSD) present at birth and other trivial lesions or variants are included [6]. A recent major meta-analysis showed that the risk of CHD in intracytoplasmic sperm injection (ICSI) and in vitro fertilization (IVF) pregnancies is significantly higher, by approximately 50%, compared to naturally conceived pregnancies [7]. Despite these findings, consensus regarding the clinical utility of performing fetal echocardiography in ART pregnancies remains elusive with conflicting recommendations provided by different scientific societies [8]. The role of ART remains uncertain and clarifying the link between ART and CHDs is of paramount importance for both clinicians and couples considering fertility treatments. We aim to determine if there is an association between CHD and different ART methods. In this paper, we present original findings from a cohort study and discuss the possible implications for clinical practice and future research.

## Materials and methods

In this retrospective cohort study, the authors selected all natural and ART pregnancies (IVF and ICSI, homologous and heterologous) referred for fetal echocardiography to IRCCS Policlinico Sant’Orsola, Bologna, between January 2005 and November 2023 diagnosed with CHD. The indications for the exams were: suspected fetal cardiac structural anomaly or abnormality of cardiac function (e.g., cardiomegaly, hydrops fetalis, irregular cardiac rhythm); fetal extracardiac anomaly; first- or second-degree relatives with CHD (parents, siblings, half-siblings); first- or second-degree relative with Mendelian inheritance disease and a history of childhood cardiac manifestations; patients diagnosed with pregestational diabetes or any type of CHD. Maternal history and characteristics, gestational age, type of ART, and CHD were retrospectively extracted from ultrasound reports. Twin pregnancies, fetuses identified with genetic anomalies via invasive diagnostic procedures or having multiple structural anomalies affecting different organs were excluded. Considering the lack of demographic and clinical data, we opted to exclude patients with a body mass index (BMI) over 35, or those with type 1 or type 2 diabetes where known. The study population was composed of two main groups: ART pregnancies diagnosed with CHD and subsequently divided according to the technique (IVF; ICSI); natural pregnancies diagnosed with CHD. In order to study the possible association of types of CHDs with ART techniques, we introduce a classification of CHDs based on the anatomical and embryological origin of the defects, which were classified into six subcategories each containing a set of anomalies: left ventricle outflow tract disease (LVOT); conotruncal abnormalities (CNTRA); valvular diseases (VD); right heart anomalies (RHA); atrioventricular septal defects (AVSD); heart rhythm diseases (HRD). Further information is shown in Table 1. Due to the low incidence, we did not evaluate situs anomalies, and anomalous pulmonary venous connection. This classification addresses the challenge of investigating specific CHDs despite limited data availability. All ultrasound cardiac assessments were carried out by a group of experienced senior operators, including fetal cardiologists and subspecialists in fetal medicine with expertise in fetal cardiac scanning. All cardiac ultrasound scans adhered to ISUOG guidelines, encompassing the 4-chamber view, outflow tracts, three vessels and tracheal view, aortic and ductal arches views, and the inferior and superior vena cava view [9, 10]. Color doppler was applied to these views, along with M-mode and spectral doppler/color doppler. The ultrasound machines used during the study period were Voluson E8 or E10 (GE Healthcare, Austria) equipped with multifrequency convex transducers. Demographic data were analyzed using routine tests. For statistical analysis, each subject in the three groups (natural, IVF, ICSI) was assigned a weight proportional to the inverse of the size of its respective group. This weighting strategy aims to allocate an equal “a priori” risk to each type of CHD across the three groups, thereby ensuring a balanced representation of subjects within each group. Multinomial logistic regression adjusted for maternal age was employed to model the relationship between each CHD category and the three groups of patients, taking into account data weighting. Under this approach, a multivariable patient-specific risk of belonging to each of the three groups is calculated for each CHD category (whether isolated or in combination). These risks are mutually exclusive, ensuring that the total risk across the three groups always sums to 100%. Comparison of the patient-specific risk distributions among and between groups was performed using ANOVA and Bonferroni post hoc test. A *p* value <0.05 was considered statistically significant. As for statistical analysis, the SPSS version 27.0 (IBM Corp. Released 2020. IBM SPSS Statistics) was adopted.

**Table 1:** Classification of CHD according to the anatomic and embryological development

Subcategory of CHD	Type of CHD
AVSD	Atrioventricular septal defect Ventricular septal defects Atrial septal defects Common atrium/cor biventricular Univentricular heart
CNTRA	Transposition of great arteries Tetralogy of fallot Double outlet right ventricle Double outlet left ventricle Truncus arteriosus
HRD	Persistent supraventricular tachycardia Atrial extrasystole Ventricular extrasystole
LVOT	Hypoplastic left syndrome disease Interruption or anomalies of aortic arch Atresia or coarctation of aorta
RHA	Hypoplastic right heart syndrome Idiopathic right heart cardiomegaly Right ventricle hypoplasia Right atrial enlargement
VD	Defects of pulmonary valves (atresia; dysplasia; stenosis; insufficiency) Ebstein’s anomaly

*AVSD* atrioventricular septal defects, *CNTRA* conotruncal abnormalities, *HRD* heart rhythm disease, *LVOT* left ventricular outflow tract abnormalities, *RHA* right heart anomalies, *VD* valvular diseases

## Results

Table 2 resumes the demographic data of the study population. As shown, maternal age was significantly higher in ART groups compared to natural pregnancies. Among the 656 cases of CHDs, the majority, 588 (89.6%), were natural pregnancies, 30 (4.6%) were conceived through IVF, and 38 (5.8%) through ICSI. All cases were categorized into six distinct groups based on anatomical and embryological criteria: AVSD (213/656, 32.5%), CNTRA diseases (219/656, 33.4%), LVOT defects (203/656, 30.9%), VD (136/656, 20.7%), RHA (26/656, 4%), and HRD (16/656, 2.4%). The most frequent combinations of CHD’s categories included AVSD + CNTRA (8.5%), AVSD + LVOT (6.3%), and AVSD + VD (5.8%). All categories’ frequencies are shown in Table 3. To comprehensively evaluate the patient-specific risk associated with each CHD’s category, we calculated the risk considering both cases where the category was isolated and those where it co-occurred with others. This approach resulted in a single patient-specific risk estimation encompassing both isolated and combined CHD’s occurrences, leading to more observations than individual cases, thus ensuring a thorough evaluation of the risk associated with each type of CHD. After adjusting for maternal age and CHD patterns, we found that the mean estimated patient-specific risk of CNTRA, AVSD, LVOT, and VD was significantly lower in IVF group compared to the natural one. The estimated mean patient-specific risk of CNTRA was higher in ICSI group versus natural pregnancies, while it was lower for AVSD and VD, and non-significant for LVOT. Conversely, the mean patient-specific risk for RHA was significantly higher both in IVF and ICSI groups compared to natural conception. HRDs were significantly more frequent in IVF versus natural pregnancies, and no cases were found in the ICSI group. When comparing ART methods, it emerged that CNTRA, VD, and RHA were more frequently observed in ICSI, while AVSD was more common in IVF. The distributions of the most prevalent association of CHD’s categories revealed that the estimated mean patient-specific risk quoted for AVSD + CNTRA, AVSD + LVOT, and AVSD + VD were more frequent in natural pregnancies. Notably, a higher mean patient-specific risk of AVSD + CNTRA was observed in ICSI vs IVF. For a summarized view of the results, refer to Table 4.

**Table 2:** Demographic data of the study population including natural, IVF, and ICSI pregnancies

Variable	Natural conceptions (*n* = 588)	IVF conceptions (*n* = 30)	ICSI conceptions (*n* = 38)	*p* value
Maternal age (mean ± SD)	32.4 ± 5.52	37.5 ± 5.60	38.4 ± 4.42	< 0.001 (α, β, δ)
Gestational age (mean ± SD)	26.5 ± 5.4	25.1 ± 5.9	24.1 ± 4.5	ns (α, δ) 0.020 (β)
Nullipara (%)	48.5	23.3	18,4	< 0.001 (α, β, δ)

*ns* non-significant

α Natural pregnancies vs IVF pregnancies; β Natural pregnancies vs ICSI pregnancies; δ IVF pregnancies vs ICSI pregnancies

**Table 3:** Frequencies of CHD’s categories among the study population

CHD’s categories	Natural conceptions (%)	IVF conceptions (%)	ICSI conceptions (%)	Total (%)
AVSD	111 (16.9)	7 (1.1)	6 (0.9)	124 (18.9)
AVSD HRD	1 (0.2)	0	0	1 (0.2)
AVSD LVOT	35 (5.3)	3 (0.5)	3 (0.5)	41 (6.3)
AVSD LVOT VD	5 (0.8)	0	0	5 (0.8)
AVSD RHA	4 (0.6)	0	0	4 (0.6)
AVSD VD	37 (5.6)	1 (0.2)	0	38 (5.8)
CNTRA	99 (15.1)	5 (0.8)	12 (1.8)	116 (17.7)
CNTRA AVSD	53 (8.1)	1 (0.2)	2 (0.3)	56 (8.5)
CNTRA AVSD HRD	1 (0.2)	0	0	1 (0.2)
CNTRA AVSD LVOT	6 (0.9)	0	0	6 (0.9)
CNTRA AVSD LVOT RHA	1 (0.2)	0	0	1 (0.2)
CNTRA AVSD RHA	1 (0.2)	0	0	1 (0.2)
CNTRA AVSD VD	6 (0.9)	0	0	6 (0.9)
CNTRA LVOT	9 (1.4)	0	0	9 (1.4)
CNTRA LVOT VD	4 (0.6)	0	0	4 (0.6)
CNTRA VD	19 (2.9)	0	0	19 (2.9)
HRD	9 (1.4)	3 (0.5)	0	12 (1.8)
LVOT	120 (18.3)	5 (0.8)	8 (1.2)	133 (20.3)
LVOT HRD	1 (0.2)	0	0	1 (0.2)
LVOT VD	3 (0.5)	0	0	3 (0.5)
RHA	8 (1.2)	2 (0.3)	0	10 (1.5)
VD	49 (7.5)	3 (0.5)	3 (0.5)	55 (8.4)
VD RHA	5 (0.8)	0	4 (0.6)	9 (1.4)
VD RHA HRD	1 (0.2)	0	0	1 (0.2)
Total	588 (89.6)	30 (4.6)	38 (5.8)	656 (100.0)

AVSD includes Atrioventricular septal defect, Ventricular septal defects, Atrial septal defects, Common atrium/cor biventricular, Univentricular Heart. CNTRA includes Transposition of great arteries, Tetralogy of Fallot, Double outlet right ventricle, Double outlet left ventricle, Truncus arteriosus. HRD includes Persistent supraventricular tachycardia, Atrial Extrasystole, Ventricular Extrasystole. LVOT includes Hypoplastic left syndrome disease, Interruption or anomalies of aortic arch, Atresia or Coarctation of aorta. RHA includes Hypoplastic right heart syndrome, Idiopathic right heart cardiomegaly, Right ventricle hypoplasia, Right atrial enlargement. VD includes Defects of pulmonary valves (atresia; dysplasia; stenosis; insufficiency), Ebstein’s anomaly

*AVSD* atrioventricular septal defects, *CHD* congenital Heart Disease, *CNTRA* conotruncal abnormalities, *LVOT* left ventricular outflow tract abnormalities, *RHA* right heart anomalies, *VD* valvular diseases, *HRD* heart rhythm disease

**Table 4:** Mean estimated patient-specific risk (%) and interquartile range of each CHD when occurring alone or in combination, calculated using weighted multinomial logistic regression across the three study groups

CHD (*n*)	Natural conceptions (*n* = 588)	IVF conceptions (*n* = 30)	ICSI conceptions (*n* = 38)	*p* value* (*α)	*p* value* (*β)	*p* value* (*δ)
CNTRA (219)	37.29 (25–49)	22.04 (18–25)	40.67 (29–48)	< 0.001	0.011	< 0.001
AVSD (213)	39.15 (18–59)	35.28 (19–51)	25.57 (22–30)	0.036	< 0.001	< 0.001
LVOT (203)	35.99 (22–52)	30.67 (25–36)	33.35 (25–40)	0.001	0.202	0.189
VD (136)	40.08 (25–47)	25.14 (23–29)	34.79 (24–41)	< 0.001	0.046	< 0.001
RHA (26)	16.50 (4–23)	32.34 (24–42)	51.16 (50–53)	< 0.001	< 0.001	< 0.001
HRD (16)	18.11 (10–20)	81.88 (75–89)	–	< 0.001	–	–
AVSD + CNTRA (56)	62.75 (54–69)	14.85 (12–18)	22.39 (12–78)	< 0.001	< 0.001	0.002
AVSD + LVOT (41)	58.14 (48–61)	22.03 (19–28)	19.83 (18–24)	< 0.001	< 0.001	0.734
AVSD + VD (38)	66.52 (47–75)	17.01 (12–27)	16.47 (13–24)	< 0.001	< 0.001	> 0.99

AVSD includes Atrioventricular septal defect, Ventricular septal defects, Atrial septal defects, Common atrium/cor biventricular, Univentricular Heart. CNTRA includes Transposition of great arteries, Tetralogy of Fallot, Double outlet right ventricle, Double outlet left ventricle, Truncus arteriosus. HRD includes Persistent supraventricular tachycardia, Atrial Extrasystole, Ventricular Extrasystole. LVOT includes Hypoplastic left syndrome disease, Interruption or anomalies of aortic arch, Atresia or Coarctation of aorta. RHA includes Hypoplastic right heart syndrome, Idiopathic right heart cardiomegaly, Right ventricle hypoplasia, Right atrial enlargement. VD includes Defects of pulmonary valves (atresia; dysplasia; stenosis; insufficiency), Ebstein’s anomaly

ANOVA and Bonferroni post hoc tests were conducted, assuming an equal number of observations among the three groups, as available for the natural group. Due to the occurrence of multiple CHDs in the same patient, the number of observations exceeds the number of cases. Quantitative maternal age was used as covariate. Only most frequent combination are reported

*AVSD* atrioventricular septal defects, *CHD* congenital Heart Disease, *CNTRA* conotruncal abnormalities, *LVOT* left ventricular outflow tract abnormalities, *RHA* right heart anomalies, *VD* valvular diseases, *HRD* heart rhythm disease

α Natural pregnancies vs IVF pregnancies; β Natural pregnancies vs ICSI pregnancies; δ IVF pregnancies vs ICSI pregnancies

## Discussion

Over the past few decades, the use of ART has substantially increased, providing a chance to conceive to many couples struggling with infertility. However, emerging evidence suggests a potential link between ART and an increased risk of fetal CHDs [11]. While this relative risk increase might appear concerning, it is crucial to recognize that ART is a highly beneficial procedure, allowing reproduction for many infertile couples who otherwise might not be able to conceive. Thus, it is essential to implement a rational management strategy for these pregnancies, such as recommending a fetal cardiac scan. This information should be used to optimize prenatal care. Indeed, most experts now agree on this association, and many scientific societies endorse fetal echocardiography in ART pregnancies. However, there is no universal consensus on this practice yet. Our study confirms very different rates of CHD categories in ART-conceived pregnancies compared to natural conception. Pregnancies achieved through ART are also associated with an increased risk of LBW [5]. Furthermore, pregnancies with major fetal CHD are significantly linked to higher risks of preeclampsia, small for gestational age, and pre-term birth [12]. Particularly, cyanotic CHD carry a higher risk of fetal growth restriction [13]. These factors can complicate the prenatal diagnosis and postnatal management of CHD. Potiris et al. conducted a comprehensive review of published literature to assess the association between ART and adverse perinatal outcomes. While they highlighted a higher risk of congenital and chromosomal defects in ART pregnancies, particularly male urogenital defects and CHD, the authors concluded that ART procedures are generally safe [14]. Conversely, Shechter-Maor et al. conducted an assessment of birth anomalies among all live births in the United States and observed that infants conceived through ART faced a twofold higher risk of experiencing one of the evaluated birth defects, with CHDs being particularly prevalent in the ART group [15]. Moreover, Talebi et al. revealed a higher occurrence not only of all types of CHDs but also of endothelial dysfunction, morphological changes, and increased vascular stiffness in infants conceived through ART compared to those naturally conceived, which is indicative of a heightened risk for premature cardiovascular issues. Consequently, the authors emphasized the necessity for increased monitoring through fetal echocardiography and postnatal cardiovascular assessment [16]. Nevertheless, the precise mechanisms linking ART and CHD remain unclear, with proposed hypotheses including the influence of underlying parental factors, such as advanced maternal age, maternal co-morbidities, and parental subfertility, which are more prevalent among couples undergoing ART procedures [7]. The pathogenesis of CHD itself remains a highly debated topic, encompassing genetic causes, environmental factors (i.e., exposure to solvents or herbicides), maternal age over 40, paternal age over 35, maternal co-morbidities (e.g., obesity, diabetes, phenylketonuria, rubella infection) and behavioral factors (i.e., alcohol, drugs, smoking) [11]. There can also be a multifactorial etiology, suggesting interaction between genetics and environmental factors [17]. Interestingly, women undergoing ART are educated about maintaining proper weight, abstaining from smoking and alcohol, suspending potentially harmful therapies, and early integration of folic acid. Therefore, the ART population is complex to investigate due to its dual nature: exposed to certain major risks (e.g., older age, subfertility, co-morbidities like diabetes and hypertension) while protected from others (e.g., obesity, smoking, alcohol, lack of folates) [15]. Despite potential confounding factors, the manipulation of gametes and embryos during ART procedures, alongside hormonal stimulation protocols, may impact embryonic development and contribute to the higher risk of CHDs [11]. Interestingly, Cortessis et al. highlighted a significant association between ART and various imprinting disorders (Angelman syndrome OR = 4.7; Beckwith–Wiedemann syndrome OR = 5.8; Prader–Willi syndrome OR = 2.2; Silver–Russell syndrome OR = 11.3), suggesting that ART could influence genetic and epigenetic mechanisms involved in fetal development [18]. As for the association between ART and congenital anomalies, contrasting evidence exists. Anzola et al. found no differences in congenital anomalies prevalence between babies conceived by fresh versus frozen embryo transfer after IVF [19]. Moreover, recent studies have attempted to synthesize data from available literature. Gullo et al. indicated an increased risk of minor CHDs among ART-conceived pregnancies compared to natural ones [11]. Conversely, Shamshirsaz et al. discovered a significantly higher risk for cyanotic congenital heart diseases (cCHDs) among infants conceived through ART (adjusted relative risk (aRR) 2.4, 95% CI 2.1–2.7) and non-ART fertility treatments (aRR 1.9, 95% CI 1.6–2.2) compared to naturally conceived infants [20]. In addition, contrasting data come from a cohort population study conducted by Iwashima et al., in which no significant differences were detected through echocardiography screening in the prevalence of CHD and severe CHD between women who conceived naturally and those who underwent ART [21]. A recent meta-analysis by Giorgione et al., indicates that the likelihood of CHDs is elevated in ART pregnancies compared to those conceived naturally (OR = 1.45). However, when analyzing the qualitative distribution of CHD types, the heightened risk was statistically significant only for smaller CHDs like VSD [7]. This was also confirmed by Aderibigbe et al. in their study, where a prevalence of minor cardiac disorders, particularly VSD, is reported among pregnancies conceived through ART [22]. At the current state, the association with major CHDs remains a point of contention, with inconsistent findings reported across various studies. Given the heterogeneous nature of these conditions, it’s crucial to consider how different subtypes of CHDs are associated with ART in order to better understand the potential implications of ART procedures on fetal cardiac development. Tararbit et al., drawing from the Paris registry of congenital malformations, identified a heightened risk for certain CHDs among fetuses conceived through IVF and ICSI, independently of chromosomal abnormalities [23]. The study suggests a stronger association of CHDs with ICSI compared to IVF and emphasizes that the choice of CHD classification can influence study outcomes, prompting the suggestion to utilize an embryological classification for analyzing the etiopathological role of ART in CHD development. They also examined increased risk in major CHD, signaling a specific rise in tetralogy of fallot (TOF) cases. ART was associated with a 2.4-fold higher risk of TOF after adjustment for maternal age, occupation, geographic origin, paternal age and year of birth; ICSI was specifically associated with a threefold higher risk of TOF. This has led to an etiopathogenetic hypothesis implicating an altered neural crest cell development in conotruncal heart defects in ART pregnancies, particularly ICSI-conceived [24]. A subsequent retrospective cohort study conducted by Galdini et al. highlighted an increased prevalence of major CHD in fetuses conceived through ART compared to natural pregnancies, notably TOF and Hypoplastic Left Heart Syndrome. However, when considering specific techniques, it presents conflicting data showing a stronger association with IVF rather than ICSI [25]. Along with the literature, our study identifies ART as a risk factor for CHD. We found that isolated RHA prevails both in IVF and ICSI pregnancies when compared to natural conception. HRD are more frequent in IVF pregnancies versus natural conception, though none data was available for ICSI pregnancies. Lastly, isolated CNTRA are more prevalent in ICSI pregnancies compared to natural conception. When comparing ART methods, it emerges a higher rate of isolated AVSD in IVF-conceived pregnancies, though we must precise that AVSD category also included minor septal defects. Whereas, CNTRA defects isolated and in combination with AVSD, VD and RHA appear to be more frequent in ICSI-conceived pregnancies. According to our results, taking into account that ART implies a major risk of cardiac anomalies, the type of ART technique plays a fundamental role in defining the severity and type of CHD. Despite advances in prenatal diagnosis, the medical and surgical management of infants with CHD still carries high risks of morbidity and mortality, especially for severe cases. Early detection through fetal echocardiography allows for timely interventions and informed decision-making regarding perinatal management. It provides the opportunity to plan deliveries at specialized centers equipped to handle complex cardiac conditions, thus improving neonatal outcomes. The higher rate of CHDs in ART pregnancies, combined with the benefits of early diagnosis and intervention, underscores the need to evaluate for routine fetal echocardiography in all ART-conceived fetuses. Overall, while the existing literature provides valuable insights into the association between ART and CHDs, discrepancies and uncertainties remain because strong levels of evidence are missing, and more prospective and case-control studies are needed. Indeed, guidelines are divergent regarding the indication to refer all ART-conceived pregnancies for an echocardiography. The American Heart Association (AHA) guidelines recognize IVF/ICSI conception as a maternal indication for fetal echocardiography with a recommendation class/level of evidence IIa/A estimating an absolute risk of 1.1–3.3% among live births [26]. Evenly, the International Society of Ultrasound in Obstetrics & Gynecology (ISUOG) [9] recognize ART conception, both IVF and ICSI, as a possible indication for fetal echocardiogram, whereas the American Society of Echocardiography (ASE) considers only IVF as a possible indication [27]. The National Guidelines for Ultrasound in Obstetrics and Gynecology published by Società Italiana di Ecografia Ostetrica e Ginecologica (SIEOG) states that considered the still unclear association between major CHD and ICSI, ICSI is not to be considered an indication for carrying out fetal echocardiography [28]. In addition, according to the analysis of Chung et al., the cost-effective method of screening for CHDs in IVF or ICSI-conceived pregnancies, is to perform a fetal echocardiogram only when a cardiac anomaly is noted during a detailed anatomy ultrasound [29].

We report the experience of one of the largest tertiary-care centers in northern Italy. The main limitation of our study is the retrospective design, anyway, considering the rarity of these conditions a prospective study would result very difficult. Plus, although the relationship between obesity and CHD is well-established in literature [30], we were unable to analyze in our study the effect of BMI on CHD due to the limited availability of data. Further research, including large-scale multicentric prospective studies, is essential to elucidate the underlying factors contributing to CHD development in ART-conceived offspring and to guide clinical decision-making and counseling for couples undergoing ART procedures.

## Conclusion

Our research findings indicate noteworthy differences in the occurrence of CHDs between pregnancies conceived through ART and those conceived naturally. Additionally, we demonstrated distinct prevalence of specific CHD categories among different ART methods, highlighting a stronger association of CHD with ICSI compared to IVF, particularly with a higher prevalence of severe CHDs like conotruncal anomalies in ICSI-conceived pregnancies. In conclusion, further research is essential to understand the impact of ART procedures on fetal cardiac development, considering the diverse range of CHD subtypes associated with ART, and to clarify the clinical implications of these associations.

## Data Availability

The data that support the findings of this study are available from the corresponding author, [LP, AF], upon reasonable request.
